# Supplementary figures and images for: Small target detection algorithm based on multi-branch stacking and new sampling transition module
Source: PLoS One. 2024 Jul 19;19(7):e0305260. doi: 10.1371/journal.pone.0305260 (PMC11259291; doi:10.1371/journal.pone.0305260)

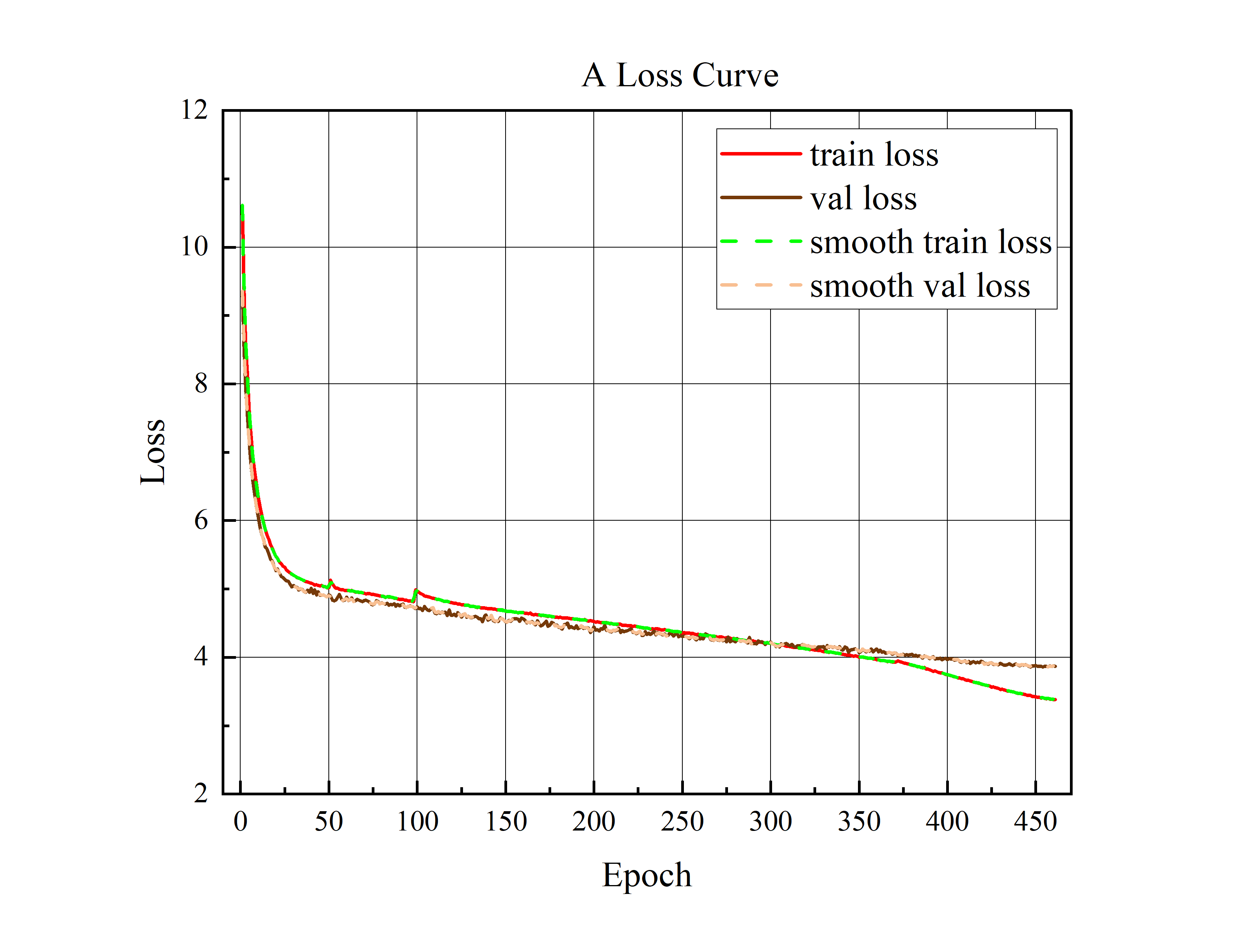

Supplement: S1 File — (ZIP) [file pone.0305260.s001.zip › coco loss.tif]

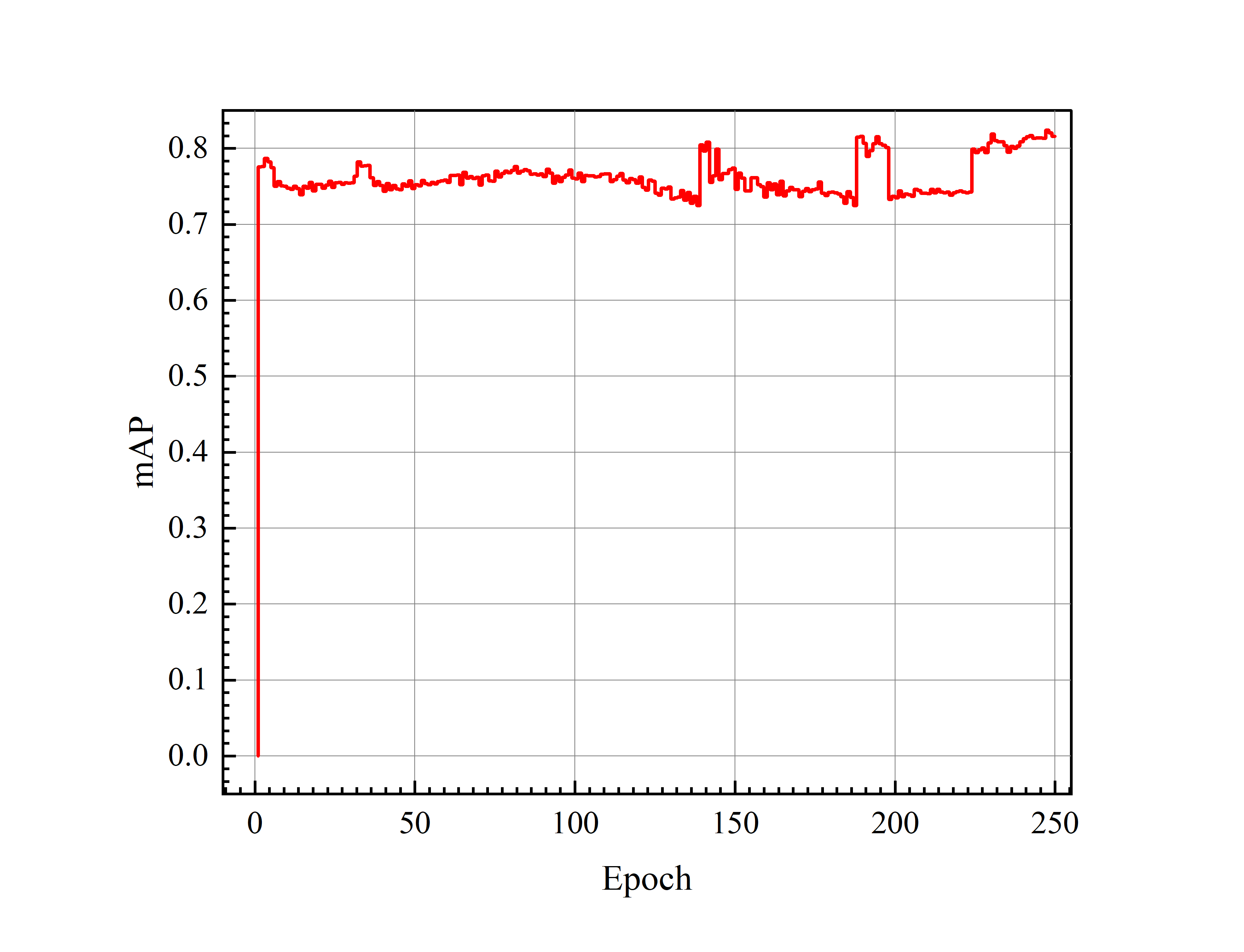

Supplement: S1 File — (ZIP) [file pone.0305260.s001.zip › mAP.tif]

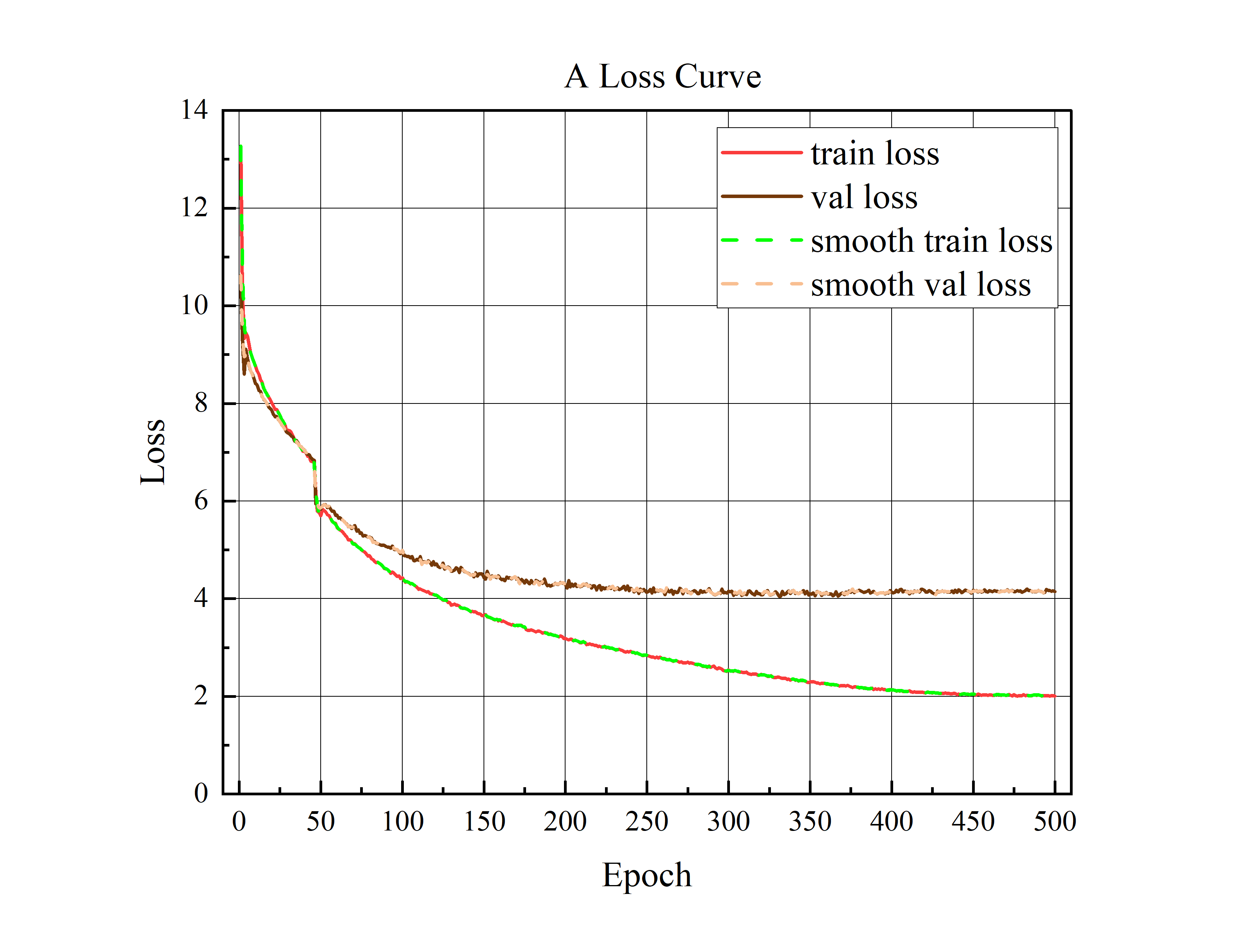

Supplement: S1 File — (ZIP) [file pone.0305260.s001.zip › voc loss.tif]
