# Supplementary material for: Small target detection algorithm based on multi-branch stacking and new sampling transition module
Source: PLoS One. 2024 Jul 19;19(7):e0305260. doi: 10.1371/journal.pone.0305260 (PMC11259291; doi:10.1371/journal.pone.0305260)
Supplement: Attachment — Submitted filename: Responce to reviewers(PONE-D-23-42468).docx [file pone.0305260.s002.docx]

Dear Editor:

Re: Manuscript (PONE-D-23-42468) – “Small target detection algorithm based on multi-branch stacking and new sampling transition module.”, by Qingyao Lin, Rugang Wang, Yuanyuan Wang, Feng Zhou.

Thank you very much for your kind consideration of our manuscript. We have carefully studied the comments raised by the reviewers and have made amendments in accordance with the comments and suggestions from the reviewers. All the changes made in the revised version are underlined. Our responses to the reviewers’ comments and suggestions are enclosed in this letter.

We look forward to hearing a favorable reply from you.

Yours sincerely,

Qingyao Lin

**Reply to Reviewer 1**

Dear Reviewer,

We would like to express our sincere thanks to the reviewer for his/her valuable comments and suggestions. We have revised the manuscript in accordance with the reviewer’s comments and suggestions. All the changes made in the revision are underlined. Our replies to the reviewer’s comments and suggestions are as follows.

**Comment #1-1:**

My big concern is the “discussion” part. I couldn’t see any discussion. In discussion, what I expect to see is that what are the plausible reasons behind obtaining these results? Moreover, the validity and accuracy of the data should be discussed. The authors must comment on whether or not the results were expected and present explanations for the results; they must go into greater depth when explaining findings that were unexpected or especially profound. If appropriate, note any unusual or unanticipated patterns or trends that emerged from your results and explain their meaning. Be sure to advocate for your findings and underline how your results significantly in move the field forward. Remember to make sure you give your results their due and not undermine them. Moreover, in discussion, you should clearly state what your study adds to the body of the literature. In discussion, the authors must explain why these results were obtained? What is/are the plausible reasons behind obtaining these results? In addition, in discussion, the authors must contrast the results of your study with those have been previously done.

Conclusion is not enough. It is not to the point. The conclusion must help the reader understand why your research should matter to them after they have finished reading the paper. A conclusion is not merely a summary of your points or a re-statement of your research problem but a synthesis of key points.

**Response #1-1:**

The reviewer’s comment is appreciated.

We agree with the reviewer's view that the discussion section is missing. It will be supplemented by an explanation of the role of the CAM module and the MSB and the reasons for this in the ablation experiment section in chapter 4.2. The discussion of the STB module and why we chose inverse subpixel convolution is placed below Table 4, which we will highlight in red in the original text. The specifics of the additions we made are as follows and will be highlighted in red in the manuscript.

From the experimental results, it can be seen that the CAM proposed in this paper is effective. In this regard, we believe that it is the ability of this structure to attend to feature channels and spatial information in a self-learning manner. Useless information can be removed by this structure and interference is minimized to improve the efficiency of the algorithm, which is in line with our original idea. We believe that the target detection task in this paper does not involve stereo targets too much, so we focus more on the sensitivity of the algorithm to channel information. This is the reason for the relatively high number of operations regarding channel feature extraction in the CAM structure. In this structure, by detecting beneficial features in the channel, which is the strength of the current feature response that is weighted and evaluated. This weight is utilized to judge the importance of features and suppress or enhance them. And the presence of pooling operations in the structure, either global average pooling or global maximum pooling, uninterruptedly extracts for the algorithm the features with the most drastic expression within a certain range; and at the same time, the scale of the feature map is reduced, which captures a large range of feature information for the algorithm. The purpose of focusing on spatial information is to enhance the network's representation of the characteristics of different spatial locations, utilizing the spatial relationships between different pixel points so that the network can better focus on spatial locations that are beneficial to the task at hand. The CAM proposed in this paper can do an effective balance between channel and spatial information, which makes the CAM perform well.

The effectiveness of MSB we believe is contributed by the multiple branches of the structure, each of which has a convolutional layer with a different convolutional kernel to extract features. If a single branch is used to perform extraction operations on features, this inevitably produces feature loss, which is naturally compensated by the structure of the MSB. Each branch has a different convolutional kernel, which makes the features extracted from each branch different and effective. The operation of stacking feature layers in MSB is to provide a guarantee that the algorithm does not lose feature information.
