# Supplementary material for: Small target detection algorithm based on multi-branch stacking and new sampling transition module
Source: PLoS One. 2024 Jul 19;19(7):e0305260. doi: 10.1371/journal.pone.0305260 (PMC11259291; doi:10.1371/journal.pone.0305260)
Supplement: Attachment — Submitted filename: Responce to reviewers(PONE-D-23-42468R1).docx [file pone.0305260.s003.docx]

Dear Editor:

Re: Manuscript (PONE-D-23-42468R1) – “Small target detection algorithm based on multi-branch stacking and new sampling transition module.”, by Qingyao Lin, Rugang Wang, Yuanyuan Wang, Feng Zhou.

Thank you very much for your kind consideration of our manuscript. We have carefully studied the comments raised by the reviewers and have made amendments in accordance with the comments and suggestions from the reviewers. All changes in the revised version will be highlighted in red. Our responses to the reviewers’ comments and suggestions are enclosed in this letter.

We look forward to hearing a favorable reply from you.

Yours sincerely,

Qingyao Lin

**Reply to Reviewer 2**

Dear Reviewer,

We would like to express our sincere thanks to the reviewer for his/her valuable comments and suggestions. We have revised the manuscript in accordance with the reviewer’s comments and suggestions. All the changes made in the revision are underlined. Our replies to the reviewer’s comments and suggestions are as follows.

**Comment #2-1:**

The motivations of the paper are not clear. The disadvantages of the existing schemes must be mentioned.

**Response #2-1:**

The reviewer’s comment is appreciated. The motivation of this paper is to address the problem that the SSD algorithm does not adequately extract the feature information contained in each feature layer and that information is lost during the sampling process. We describe these elements in the abstract section. The disadvantage of the research in this paper is that the detection speed of the algorithm is not strictly required, thus making the algorithm only initially capable of real-time detection. We will add to the above shortcoming of the algorithm in the CONCLUSION section.

**Comment #2-2:**

Contributions are not mentioned. Most importantly, the structure of the Introduction section is very poor.

**Response #2-2:**

The reviewer’s comment is appreciated. The contribution is mainly described in the third paragraph of the introduction section. Perhaps our presentation was not clear, so we have modified this section to highlight the content of the contribution.

**Comment #2-3:**

Related schemes are not discussed properly. The following papers must be included:
Performance releaser with smart anchor learning for arbitrary-oriented object detection
Machine learning insights into hypersonics research evolution: A 21st century perspective
IFODPSO-based multi-level image segmentation scheme aided with Masi entropy
Needle detection and localisation for robot-assisted subretinal injection using deep learning
Data accessing based on the popularity value for cloud computing
Leveraging deep learning techniques to obtain efficacious segmentation results
QEST: Quantized and efficient scene text detector using deep learning

**Response #2-3:**

The reviewer’s comment is appreciated. We have discussed the above article and placed it in the INTRODUCTION section. The details are as follows:

There are many important downstream branch tasks in the field of computer vision, and target detection, as an important one, has achieved remarkable results in various fields. Zhang[1]et al. proposed an ALS-Performance Releaser (PRSAL) with the learning function of intelligent anchors for anchor frame learning strategies. It utilizes anchor classification ability as an equivalent indicator of anchor box regression ability to screen anchors with high detection potential in a more rational way. Chakraborty R[2]et al. proposed an improved version of Fractional Order Darwinian PSO (IFODPSO) for segmenting histogram based 3D color images on multiple levels of Berkeley Segmentation Data Set (BSDS500). This scheme overcomes the complete dependence on score coefficients when dealing with multilevel problems with datasets, which provides new ideas for studying computer vision segmentation tasks. Zhou M[3]et al. demonstrated a robust framework for needle detection and localization in robot-assisted subretinal injections using a cross-cutting study between deep learning and medical contexts. The best performing network successfully detected and localized all needles in the dataset with an IoU value of 0.55 when evaluated on live pig eyes. Manjari K[4]et al. developed a novel lightweighting model with ResNet50 and MobileNetV2 as the backbone, which improves the efficiency of scene text detection and reduces the resource cost. It provides ideas for our subsequent research on network lightweighting.

**Comment #2-4:**

Algorithm or step-wise discussion of the proposed scheme is not mentioned.

**Response #2-4:**

The reviewer’s comment is appreciated. The proposed scheme is based on the SSD algorithm. And the step-wise discussion we have described in the ablation experiment section.

**Comment #2-5:**

The proposed scheme is unstructured. It is hard to identify the novelty of the proposed work.

**Response #2-5:**

The reviewer’s comment is appreciated. The structural diagram of the proposed scheme we give in Fig. 2. The implementation and functionality of each module is related in Analysis of the AMT-SSD algorithm model section. Among them, MC Block and Tran Block are new modules with innovative design in SSD algorithm.

**Comment #2-6:**

Equations and figures are not represented properly. Also, the key terms of many equations are not defined.

**Response #2-6:**

The reviewer’s comment is appreciated. The formulas and numerical expressions that appear in the Composite Attention Module section represent only the implementation of the CAM module. Where equations and figures are missing from the text, we will complete them.

**Comment #2-7:**

Technical discussion on results is not given. Moreover, the results are not convincing.

**Response #2-7:**

The reviewer’s comment is appreciated. The discussion of the results we describe in the ablation experiments section. In this section we provide a detailed description of the role of each module and the reasons for the advantages achieved.

**Comment #2-8:**

The English language is very poor.

**Response #2-8:**

The reviewer’s comment is appreciated. We have embellished and revised the language.

**Comment #2-9:**

The organization of the paper is poor.

**Response #2-9:**

The reviewer’s comment is appreciated. We revised the language of the paper as well as the relevant statements and did our best to make the paper logical and well-organized.

**Comment #2-10:**

Important references are missing and all the details of the references are not given.

**Response #2-10:**

The reviewer’s comment is appreciated. Missing relevant literature we will add and elaborate in detail in the introduction section. These will be revised in tandem with Comment #2-3.

**Comment #2-11:**

Delete Author Summary.

**Response #2-11:**

The reviewer’s comment is appreciated. The Author Summary are deleted.

**Reply to Reviewer 3**

Dear Reviewer,

We would like to express our sincere thanks to the reviewer for his/her valuable comments and suggestions. We have revised the manuscript in accordance with the reviewer’s comments and suggestions. All the changes made in the revision are underlined. Our replies to the reviewer’s comments and suggestions are as follows.

**Comment #3-1:**

I couldn't find the chapter 4.2 as highlighted by the author in their comments, " the ablation experiment section in chapter 4.2.".

**Response #3-1:**

The reviewer’s comment is appreciated. I'm very sorry for the inconvenience. The contents of the ablation experiments are shown in Table 3, and the related analyses are also presented near Table 3.

**Comment #3-2:**

There are many vague and irrelevant sentences which do not convey any significant meaning: For example, "In this structure, by detecting beneficial features in the channel, which is the strength of the current feature response that is weighted and evaluated..."

**Response #3-2:**

The reviewer’s comment is appreciated. We have revised the relevant statements to ensure that a clearer meaning can be conveyed. The details are as follows:

In this structure, the strength of the beneficial features in the channel is evaluated and from this aspect, the network model is judged whether the features need to be suppressed or boosted.

**Comment #3-3:**

The structure and sentence formation is too long which is breaking the true meaning that author is trying to explain. Multiple things are explained in long statements which diverts the reader to understand properly.

**Response #3-3:**

The reviewer’s comment is appreciated. We have revised excessively long statements in the text and reorganized the narrative for simplicity and clarity.

**Comment #3-4:**

The references are written in haphazard manner not following proper coherence or uniformity.

**Response #3-4:**

The reviewer’s comment is appreciated. We would like to cover as much as possible of the research program and research background in terms of references. So the references covered mainly the development of SSD algorithms for target detection. It also contains some research content for small targets, although these research programs do not use SSD algorithms, but provide ideas for small target research.

**Comment #3-5:**

The first line of the Conclusion section: In order to solve the problem of poor feature expression and easy loss of feature information during the sampling process, resulting in poor performance in small target detection. " seems to be incomplete and over lengthy breaking the exact meaning author wants to explain.

**Response #3-5:**

The reviewer’s comment is appreciated. In response to this problem, we have made changes to reorganize and recount the statements. The details are as follows:

A small target detection algorithm AMT-SSD that combines the multi-branch stacking of the attention mechanism and the new sampling transition module proposed in this paper. And the AMT-SSD improves the detection performance of small targets while improving the target feature expression and reducing the loss of feature information during the sampling process.

**Comment #3-6:**

The way this paragraph is written: We believe that this is because compared with the down-sampling methods of maximum pooling and ordinary convolution, inverse sub-pixel convolution uses the feature information of the feature map itself to directly change the scale, rather than through convolution operations and take the maximum value to fill in the feature information" sounds very casual and informal. Again diverts the reader from exact information.

**Response #3-6:**

The reviewer’s comment is appreciated. We have revised the expression to convey the exact meaning to the readers in a more formal and rigorous way.

From the experimental results in Table 4, the inverse subpixel convolution has good feature extraction. We believe that this is due to its utilization of its own feature information to change the dimension of the feature map. Inverse subpixel convolution can reduce the size of the feature map while effectively utilizing the feature information to achieve feature scale balance.
